# Highly Sensitive Voltammetric Determination of Acrylamide Based on Ibuprofen Capped Mercury Nanoparticles

**DOI:** 10.3390/s21217302

**Published:** 2021-11-02

**Authors:** Sirajuddin, Zulfiqar Ali Tagar, Muhammad Anwar Ul Haq, Muhammad Raza Shah, Mujeeb-ur-Rehman, Syed Tufail Hussain Sherazi, Jiri Barek, Muhammad Siddique Kalhoro

**Affiliations:** 1 International Center for Chemical and Biological Sciences, HEJ Research Institute of Chemistry, University of Karachi, Karachi 75270, Pakistan; drsiraj03@yahoo.com (S.); anwarulhaq196@gmail.com (M.A.U.H.); raza.shah@iccs.edu (M.R.S.); mbtk.chem@gmail.com (M.-u.-R.); 2 National Center of Excellence in Analytical Chemistry, University of Sindh, Jamshoro 76080, Pakistan; tagarzulfiqar@gmail.com (Z.A.T.); tufail.sherazi@gmail.com (S.T.H.S.); 3 UNESCO Laboratory of Environmental Electrochemistry, Department of Analytical Chemistry, Faculty of Science, Charles University, Albertov 6, CZ-15843 Prague, Czech Republic; 4 Institute of Physics, University of Sindh, Jamshoro 76080, Pakistan; mskalhoro@hotmail.com

**Keywords:** mercury nanoparticles, ibuprofen, square wave voltammetry, acrylamide sensor, real water samples

## Abstract

Highly stable, small-sized and evenly distributed solid mercury nanoparticles capped with ibuprofen (Ibu-HgNPs) were prepared via reduction with hydrazine and capped with ibuprofen as a stabilizing agent. Characterization of Ibu-HgNPs was carried out by UV-Vis spectrophotometry and transmission electron microscopy (TEM). The prepared Ibu-HgNPs were immobilized onto a glassy carbon electrode (GCE) and used for the first time as the sensing element for voltammetric determination of low concentrations of acrylamide (AA) in aqueous solutions. Various parameters such as the type of supporting electrolyte, voltammetric mode, frequency, deposition time, stirring rate and initial potential were optimized to obtain the highest peak current of AA. The sensor delivered the best results in combination with the square wave voltammetry (SWV) mode, with good repeatability (relative standard deviation (RSD) of 25 repetitions was 1.4% for 1000 ppb AA). The study further revealed that Ibu-HgNPs are strongly adhered to GCE and hence do not contaminate the environment even after several runs. The newly developed AA sensor provides linear calibration dependence in the range of 100–1300 ppb with an R^2^ value of 0.996 and limit of detection (LOD) of 8.5 ppb. Negligible interference was confirmed from several organic compounds, cations and anions. The developed sensor was successfully applied for AA determination in various types of environmental real water samples to prove its practical usefulness and applicability.

## 1. Introduction

Acrylamide (AA) is an industrially important hydrophilic organic compound applied globally to produce polyacrylamide (PAA), which is used in various fields (e.g., as flocculent and coagulant agent, as grouting agent for water pipes, tunnels, cosmetics, plastics, paper and textile industries and in the form of a solid support for proteins’ separation via electrophoresis). Owing to hydrogen bonding, AA is easily soluble in water and hence easily movable in the environment. Release of the AA monomer from PAA in related industries, soil grouting and other situations is a major cause of water pollution. According to the International Agency for Research on Cancer (IARC), AA and its epoxide metabolites are toxic to reproductive system, neurotoxic and carcinogenic [1,2,3,4]. Several methods have been reported so far for AA determination including liquid chromatography with pulsed electrochemical detection (LC ED) [5], ion exclusion liquid chromatography coupled with diode array detection (LC–DAD) [6], gas chromatography with electron capture detector (GC-ECD) [7], liquid chromatography coupled with tandem mass-spectrometry (LC–MS/MS) [8], liquid chromatography-mass spectrometry (LC-MS) [9], combination of ion-exclusion chromatographic separation and MS detection [10], reversed-phase high-performance liquid chromatography (RP-HPLC) [11], normal phase HPLC [12], HPLC MS-MS [13], colorimetry [14] and fluorescence spectroscopy [15]. However, voltammetric methods are considered the most economical, simple and sensitive, based on portable and inexpensive instrumentation and a simple redox reaction, and they provide a high sensitivity and reasonable selectivity. Catalytic square wave voltammetry at a hanging mercury drop electrode [16] is a good example with a limit of detection (LOD) around 4 ppb. Niaz et al. developed a differential pulse polarographic (DPP) method for the determination of AA directly in a neutral 0.05 mol L^−1^ LiCl aqueous solution using a dropping mercury electrode (DME). At optimum conditions, the calibration graph was linear in the concentration range from 0.2 ppm to 20 ppm [17]. Due to fear of mercury toxicity, attempts have been made to use various modified electrodes to make the electrochemical method more economical, sensitive and environmentally friendly. For instance, single-walled carbon nanotubes (SWCNTs) and hemoglobin (Hb)-modified glassy carbon electrodes (GCE) have been used for voltammetric detection of AA with very low LOD 1.0 ppb in aqueous extracts from potato crisps [18]. An imprinted sol-gel polymer on an AuNPs-MWCNTs-CS modified electrode [19] and hemoglobin-oligonucleotides-modified electrode [20] was also successfully used for the determination of AA.

**Figure 1:**
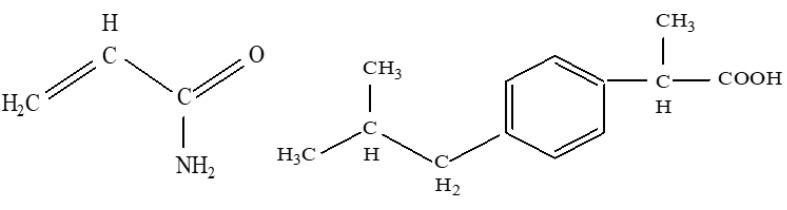
Structural formulas of acrylamide (**left**) and ibuprofen (**right**).

In the present paper, we use another novel strategy for electrode modification. We synthesize mercury nanoparticles (HgNPs) via reduction of Hg^2+^ ions by hydrazine, capping them with ibuprofen (see Figure 1) as the stabilizing agent. These HgNPs were deposited on the surface of GCE and applied as a highly sensitive and selective principally novel type of voltammetric modifier for AA detection in various types of water samples.

## 2. Materials and Methods

### 2.1. Reagents and Materials

Mercuric chloride (HgCl_2_), sodium hydroxide (NaOH), hydrazine (N_2_H_2_), Nafion^TM^, ibuprofen, potassium nitrate (KNO_3_), sodium chloride (NaCl), calcium nitrate (Ca(NO_3_)_2_), ammonium nitrate (NH_4_NO_3_), acrylamide (electrophoresis grade) and lithium chloride (LiCl) (all analytical grade) were obtained from E. Merck; tetramethylammonium bromide (TMAB), tetramethylammonium iodide (TMAI) and tetrabutylammonium iodide (TBAI) from Fluka; nitric acid (HNO_3_), acetone, acrolein, acrylic acid, maleic acid, glucose, citric acid and starch from BDH, and potassium chloride from Sigma-Aldrich. Stock solution of 0.5% ibuprofen was prepared in methanol. 0.5% Nafion^TM^ solution was prepared in *iso*-propanol via intensive sonication. Stock solutions of 0.02 mol L^−1^ HgCl_2_, 0.4 mol L^−1^ NaOH, 0.05 mol L^−1^ LiCl, TMAB, TBAB, TMAI and TBAI and all other salts solutions were prepared in milli Q^®^ deionized water (100 µS/cm).

### 2.2. Apparatus

Ibu-HgNPs were characterized via UV-Vis spectrophotometry (Lambda 2) of Perkin Elmer in the range of 200–800 nm. Scanning transmission electron microscopy (STEM) imaging was carried out using a model TECNAI F300 electron microscope (FEI Company) working at an accelerating power of 200 kV, while voltammetric study was performed by a VA 797 Trace Analyzer from Metrohm, Switzerland using Ibu-HgNPs-GCE as a working electrode, Ag|AgCl (3.5 mol L^−1^ KCl) as a reference electrode and Pt rod as a counter electrode (see Section 2.5).

### 2.3. Synthesis of Ibu-HgNPs

Ibu-HgNPs were synthesized according to [21]. Briefly, 1.2 mL of 0.02 mol L^−1^ HgCl_2_ solution was diluted to 10 mL with milli Q**^®^** water and its pH was adjusted to 1.5 by adding 1 mol L^−1^ HCl. To this solution, 90 µL of 0.4 mol L^−1^ NaOH was added followed by 150 µL of 0.1 mol L^−1^ hydrazine and 400 µL of 0.5% ibuprofen solution in the mentioned order with the final adjustment to 20 mL by milli Q**^®^** water. The solution was mixed by stirring with a glass rod for 1.5 min until a slightly whitish milky solution was formed with a pH of 4.0 ± 0.1.

### 2.4. STEM Characterization of HgNPs

Samples for STEM characterization were prepared by placing a drop of Ibu-HgNPs solution prepared by the above-described procedure on a carbon-coated copper grid and drying under a vacuum at room temperature.

### 2.5. Cleaning and Modification of Glassy Carbon Electrode

A glassy carbon electrode (GCE, 3 mm diameter) was cleaned first by polishing with aqueous paste of 0.05 µm alumina followed by sonication in milli Q**^®^** water for 5 min and drying under pure nitrogen. A 5 µL drop of 0.5% Nafion^TM^ solution was deposited on the surface of GCE and dried with a hair dryer, and another similar drop was applied over GCE and dried again in the same way. Afterwards, a 5 µL drop of Ibu-HgNPs was placed over the layer of Nafion^TM^ treated GCE and dried in the same way as in the case of Nafion^TM^ deposition. Afterwards, the prepared modified GCE further denoted as Ibu-HgNPs-GCE was used as a working electrode for AA detection in all further studies.

### 2.6. Voltammetric Studies

Cyclic voltammetry (CV), square wave voltammetry (SWV) and differential pulse voltammetry (DPV) were used in following voltammetric studies. A three-electrode arrangement was used with Ibu-HgNPs-GCE as the working electrode, Ag|AgCl (3.5 mol L^−1^ KCl) as the reference electrode and a Pt rod as the counter electrode. In this study, 20 mL 0.05 mol L^−1^ LiCl mixed with 0.05 mol L^−1^ TMAB in an 8:2 *v*/*v* ratio (pH 6.9 ± 0.1) was used as a supporting electrolyte unless stated otherwise. Nitrogen purging for 240 s was used to remove dissolved oxygen. For the CV scan rate 100 mVs^−1^ was used unless stated otherwise. SWV in the potential range from −0.1 to −2.0 V with a frequency of 50 Hz was used unless stated otherwise. For DPV, the following parameters were used unless stated otherwise: scan rate 20 mVs^−1^, pulse amplitude 50 mV, pulse width 100 ms.

### 2.7. Application of Ibu-HgNPs-GCE to Tap Water Samples and River Indus Water Samples

The tap water sample was collected from the Electrochemistry and Nanotechnology Laboratory building at the University of Sindh, while river water samples were collected from three different sites of River Indus: Hyderabad Division, Sindh, Pakistan. For the research, 10 mL of 0.05 mol L^−1^ LiCl and 0.05 mol L^−1^ TMAB mixture (8:2 *v*/*v*) in deionized water was made to 20 mL with tap water containing 200 ppb, 500 ppb and 700 ppb of AA. Each sample was analyzed in triplicate using parameters described in Section 2.6. Samples collected from the River Indus were first filtered using Whatman filter paper No. 1 and then handled in the same way as the tap water samples.

## 3. Results and Discussion

### 3.1. Characterization of Modified Electrode (Ibu-HgNPs-GCE)

#### 3.1.1. UV-Vis Spectrophotometry

**Figure 2:**
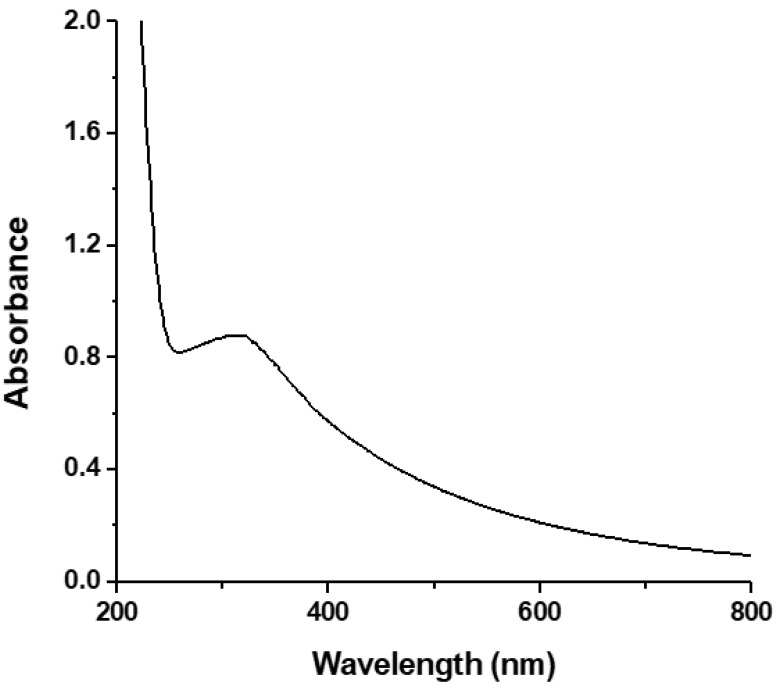
UV-Vis spectrum of Ibu-HgNPs prepared under optimized conditions stated in Section 2.3 using milli Q**^®^** water as blank. Optical path length 10 mm.

The UV-Vis spectrum of HgNPs, formed using hydrazine as a reducing agent and ibuprofen as a capping agent, is depicted in Figure 2.

This UV-Vis spectrum corresponds to a previously published one [19] and confirms the formation of ibuprofen capped HgNPs under these conditions.

#### 3.1.2. TEM Characterization of HgNPs

**Figure 3:**
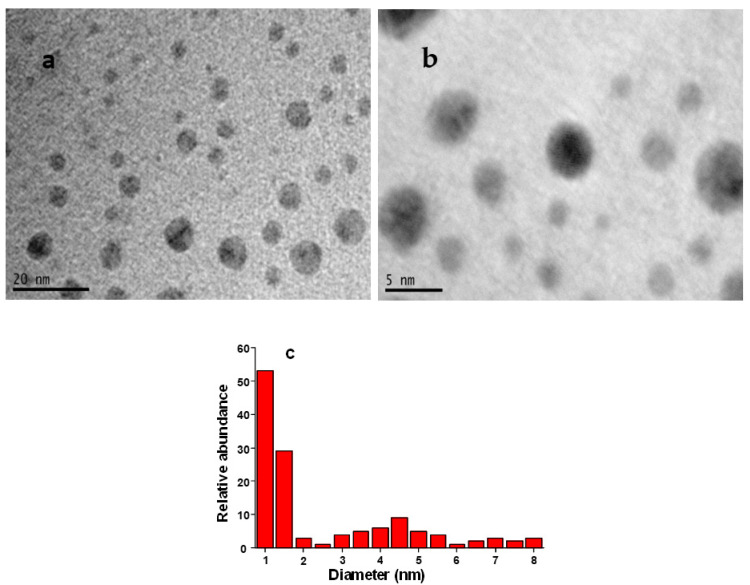
TEM images (**a**,**b**) and size distribution chart (**c**) of prepared IbuHgNPs.

Figure 3 demonstrates the TEM images along with a size distribution chart of the Ibu-HgNPs formed under optimized conditions on a carbon-coated copper grid.

**Figure 4:**
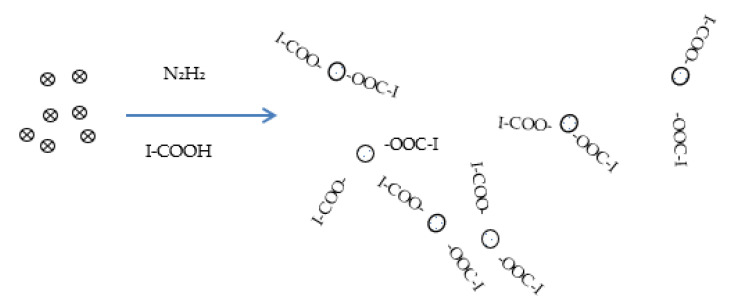
Proposed mechanism for the formation of Ibu-HgNPs in solution.

The images show the formation of broadly distributed spherical HgNPs. The size distribution chart depicts the range of HgNPs from 1–8 nm with an average diameter of 3.5 ± 1.2 nm. The small sizes of these nanoparticles indicate that they can possess good catalytic properties. These results are in agreement with previous studies [21,22]. The following mechanism for the formation of Ibu-HgNPs in solution can be proposed (Figure 4).

Here, ⊗ represents the Hg^2+^ ions in aqueous solution and I-COO-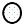 or 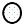-OOC-I show the interaction of ibuprofen with HgNPs, resulting in their stabilization against agglomeration. Firstly, hydrazine (N_2_H_2_) is converted into gaseous N_2_ and H_2_ where N_2_ plays the role of remover of oxygen from water, thus hindering oxygen attack on HgNPs and hence avoiding the formation of HgO, while H_2_ works as a strong reducer for the conversion of Hg^2+^ ions into HgNPs. Ibuprofen (represented as I-COOH in Figure 4) is present at the used pH in dissociated-form I-COO^-^. As soon as the HgNPs are formed via reduction by hydrazine, the ibuprofen interacts with HgNPs to keep them small and stabilized against agglomeration. The TEM images in Figure 3 prove the presence of small and ibuprofen-stabilized HgNPs.

### 3.2. Voltammetric Study

At first, optimization of various parameters of the voltammetric determination of AA via Ibu-HgNPs-GCE in aqueous system was carried out. The following parameters were optimized using 1000 ppb standard solution of AA, Ibu-HgNPs-GCE and 240 s bubbling with nitrogen to remove oxygen.

#### 3.2.1. Effect of Different Supporting Electrolytes

**Figure 5:**
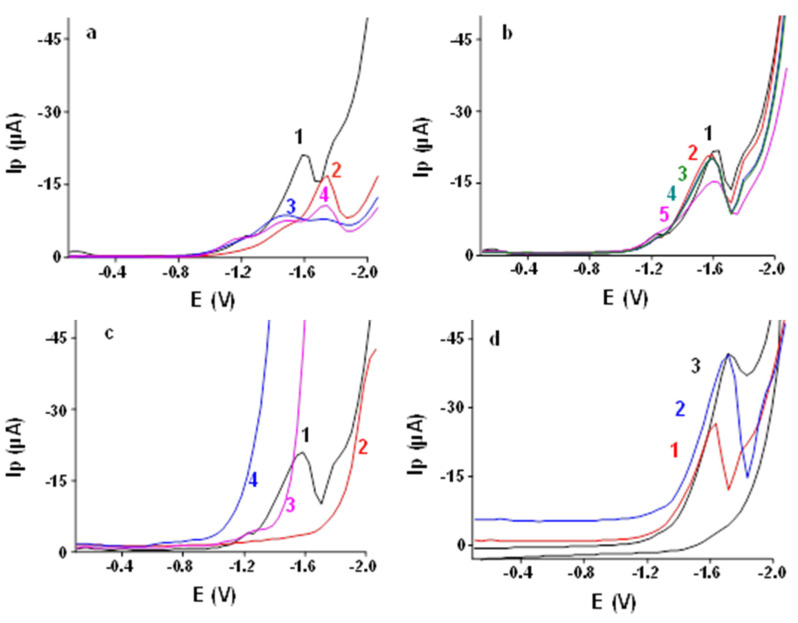
DP voltammograms of 1000 ppb AA at Ibu-HgNPs-GCE, (**a**) Effect of different supporting electrolytes, (1) TMAB, (2) LiCl, (3) TMAI, (4) TBAI (each 0.05 mol L^−1^); (**b**) Effect of different ratios of LiCl:TMAB (each 0.05 mol L^−1^), (1) 9:1, (2) 8:2, (3) 7:3, (4) 6:4, (5) 5:5 TBAI; (**c**) Effect of different working electrodes, (1) Ibu-HgNPs-GCE, (2) bare GCE, (3) bare AgE, (4) bare AuE; (**d**) Effect of different voltammetric techniques, (1) DPV, (2) SWV, (3) CV.

Here, 0.05 mol L^−1^ LiCl, TMAB, TMAI and TBAI were tested to determine the AA using DPV at Ibu-HgNPs-GCE in the potential range of −0.1 V to −2.0 V in aqueous solution (see Figure 5a). Tested buffers are frequently used in our laboratory with satisfactory results and thus we did not feel it was necessary to test a large number of other available buffers. The highest and best developed peaks were obtained using TMAB and LiCl. Therefore, we further tested various combinations of LiCl and TMAB.

#### 3.2.2. Effect of Ratio of LiCl to TMAB in Supporting Electrolytes

Different *v*/*v* ratios of 0.05 mol L^−1^ LiCl and TMAB (9:1, 8:2, 7:3, 6:4, 5:5) were tested using DPV at Ibu-HgNPs-GCE (Figure 5b). The best results were obtained with 0.05 mol L^−1^ LiCl to 0.05 mol L^−1^ TMAB at a *v*/*v* ratio 8:2, which was used for further optimization.

#### 3.2.3. Comparison of Different Working Electrodes

For the sake of comparison, various working electrodes (Ibu-HgNPs-GCE, bare GCE, bare silver electrode (AgE) and bare gold electrode (AuE)) were used for determination of 1000 ppb AA (Figure 5c) in 0.05 mol L^−1^ LiCl and 0.05 mol L^−1^ TMAB (*v*/*v* ratio 8:2). It was obvious that HgNPs-GCE was the best electrode for AA determination in comparison with other electrodes. Hence, Ibu-HgNPs-GCE was used for further studies.

#### 3.2.4. Comparison of Different Voltammetric Techniques

Various voltammetric techniques (CV, DPV, and SWV) were tested for determination of 1000 ppb AA on Ibu-HgNPs-GCE (see Figure 5d). CV revealed that the AA reduction on HgNPs-GCE was irreversible, which was in agreement with paper [17], which assumes irreversible reduction of AA to propionamide via two electrons’ and two protons’ transfer. However, our paper is focused on voltammetric determination of AA and thus the mechanism of its electrode reaction was not investigated in detail. Both the peak shape and height and background current were optimal in the case of SWV (in the case of DPV, the observed peak current of AA was somewhat lower). Therefore, SWV was used for further studies.

#### 3.2.5. Effect of SWV Frequency

**Figure 6:**
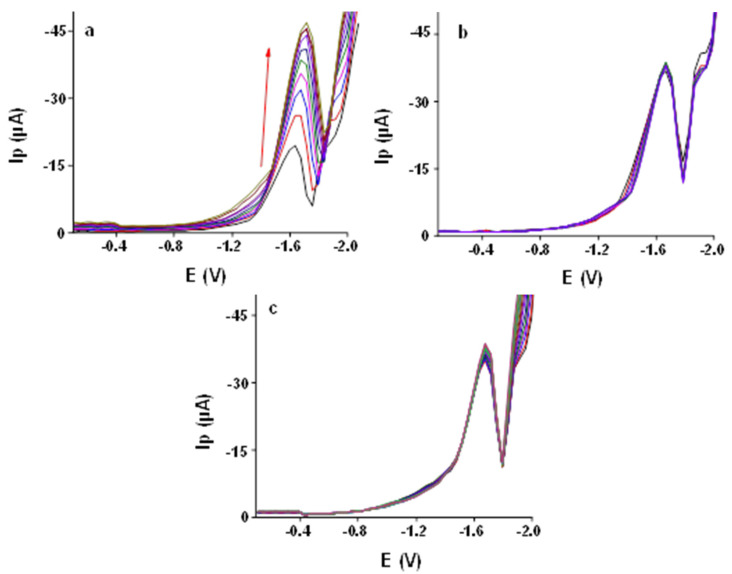
Effect of different parameters on SWV of 1000 ppb AA at Ibu-HgNPs-GCE in 0.05 mol L^−1^ LiCl and 0.05 mol L^−1^ TMAB (*v*/*v* ratio 8:2). (**a**) Various frequencies (10, 20, 30, 40, 50, 60, 70, 80, 90, 100 Hz), accumulation time 0 s, stirring rate 0 rpm. (**b**) Accumulation time from 0 to 360 *s,* stirring rate 0 rpm, frequency 50 Hz. (**c**) Stirring rate from 0 to 3000 rpm, frequency 50 Hz, accumulation time 60 s.

The influences of various SWV frequencies on the peak current of 1000 ppb AA (10, 20, 30, 40, 50, 60, 70, 80, 90 and 100 Hz) at Ibu-HgNPs-GCE were tested (see Figure 6a). It can be seen that with increasing frequency, the SWV peak current of AA increases, with a slight shift of the peak potential towards more negative values. A frequency higher than 50 Hz generates distortion and a broadening effect with an additional shoulder peak. Therefore, the 50 Hz frequency was selected for further studies.

#### 3.2.6. Effect of Accumulation Time and Stirring Rate

The effect of accumulation time from 0 to 360 s was studied using Ibu-HgNPs-GCE for 1000 ppb of AA at the deposition potential of −0.4 V (Figure 6b) and stirring rate of 1000 rpm. A negligible effect was observed on the peak current and the same held for the stirring rate during accumulation (Figure 6c). Therefore, accumulation was not used in further studies.

#### 3.2.7. Effect of Initial Potential

**Figure 7:**
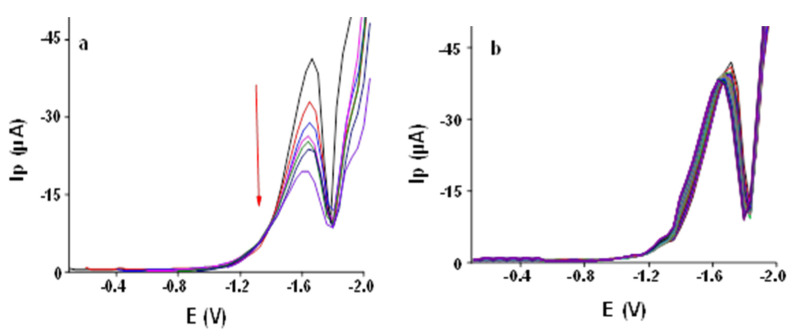
SW voltammograms (50 Hz) of 1000 ppb AA at Ibu-HgNPs-GCE in 0.05 mol L^−1^ LiCl and 0.05 mol L^−1^ TMAB (*v*/*v* ratio 8:2). (**a**) Effect of initial potential (−0.1, −0.2, −0.4, −0.6, −0.8, −1.0, −1.1 V). (**b**) Repeatability for 25 repeated voltammograms at optimum conditions given in 3.2.9.

Different initial potential values (−0.1, −0.2, −0.4, −0.6, −0.8, −1.0, −1.1 V) were applied to see their effect on the peak current of 1000 ppb AA using SWV at Ibu-HgNPs-GCE (see Figure 7a). The peak current decreased with decreasing initial potential. Hence, –0.1 V initial potential was selected as optimum for further studies.

#### 3.2.8. Repeatability Test

Twenty-five repetitive SWV of 1000 ppb AA at Ibu-HgNPs-GCE in 0.05 mol L^−1^ LiCl:TMAB (8:2 *v*/*v* ratio) are depicted in Figure 7b. The obtained relative standard deviation (RSD) of 1.4% proves the good performance and high stability of the newly developed sensor. This means that Ibu-HgNPs-GCE can be used for at least 25 consecutive measurements without appreciable decrease in response (negligible electrode passivation). Therefore, there is no need to renew the electrode surface after a few measurements. The electrode response is sufficiently stable for the whole day. If the performance of the electrode deteriorates for any reason, it can be renewed by repeating the procedure of its preparation. However, we could use the electrode for the whole day without any problem.

#### 3.2.9. Calibration Plot

**Figure 8:**
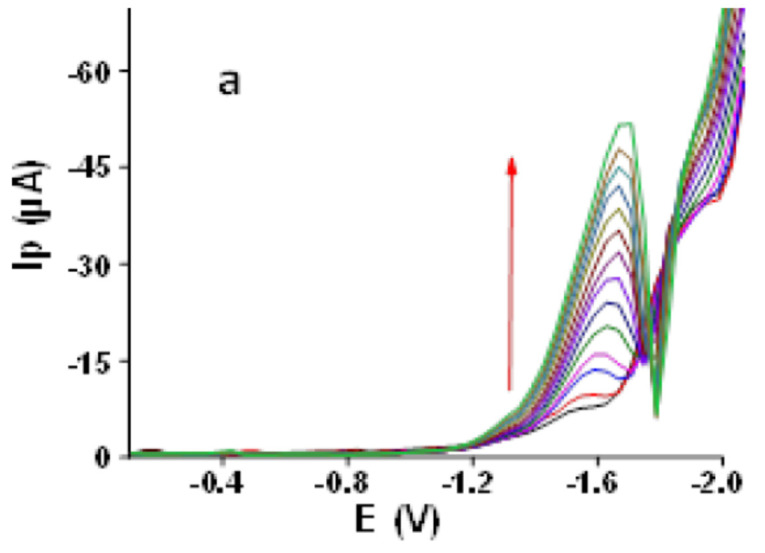

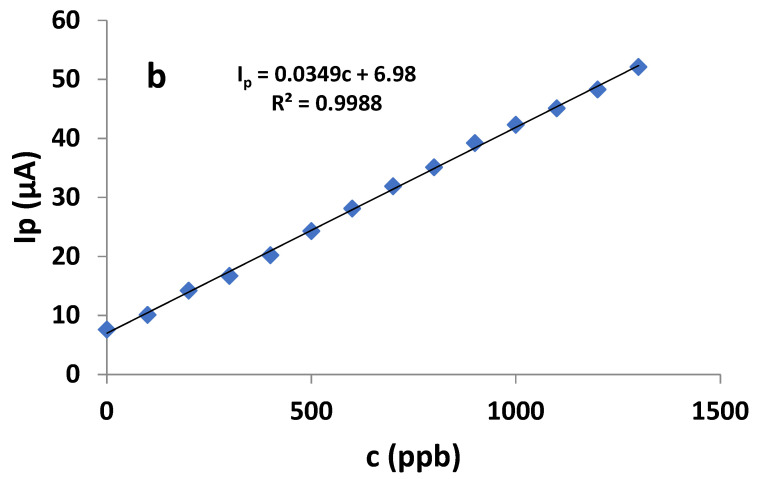
SWV (50 Hz) determination of AA at Ibu-HgNPs-GCE in 0.05 M LiCl and 0.05 M TMAB (*v*/*v* ratio 8:2). (**a**) Corresponding SW voltammograms (0 and 100–1300 ppb AA). (**b**) Corresponding linear calibration plot in the range of 0–1300 ppb AA. Optimum conditions described in Section 3.2.9.

Calibration dependence was plotted in the broad concentration range of 100–1300 ppb AA using SWV at newly developed Ibu-HgNPs-GCE under the following optimized conditions, resulting from previous experiments (higher concentrations were not investigated because they are not realistically expected in drinking water): supporting electrolyte 0.05 mol L^−1^, LiCl-0.05 mol L^−1^, TMAB *v*/*v* ratio 8:2, Ibu-HgNPs-GCE, SWV in potential range from −0.1 to −2.0 V with frequency of 50 Hz. The corresponding plot (Figure 8b) shows excellent linearity (R^2^ = 0.9988 and LOD of 8.5 ppb, 3 s/k, where s is the standard deviation of blank runs and k is the slope of linear calibration plot [17]).

#### 3.2.10. Interferences

The effect of interfering ions and compounds was studied to verify the selectivity of Ibu-HgNPs-GCE for 1000 ppb AA with a 10:1 ratio of interfering agent to AA, as shown in Table 1. It was evident that the effect of these individual interferents lay in the range of +0.5 to −3.5%, while the overall (combined) effect was equal to −3.1%. In other words, all these interferences were negligible within the acceptable range of ±5.0%. Therefore, our newly developed Ibu-HgNPs-GCE is highly selective for AA detection.

#### 3.2.11. Application of Ibu-HgNPs-GCE to Real Water Samples

**Figure 9:**
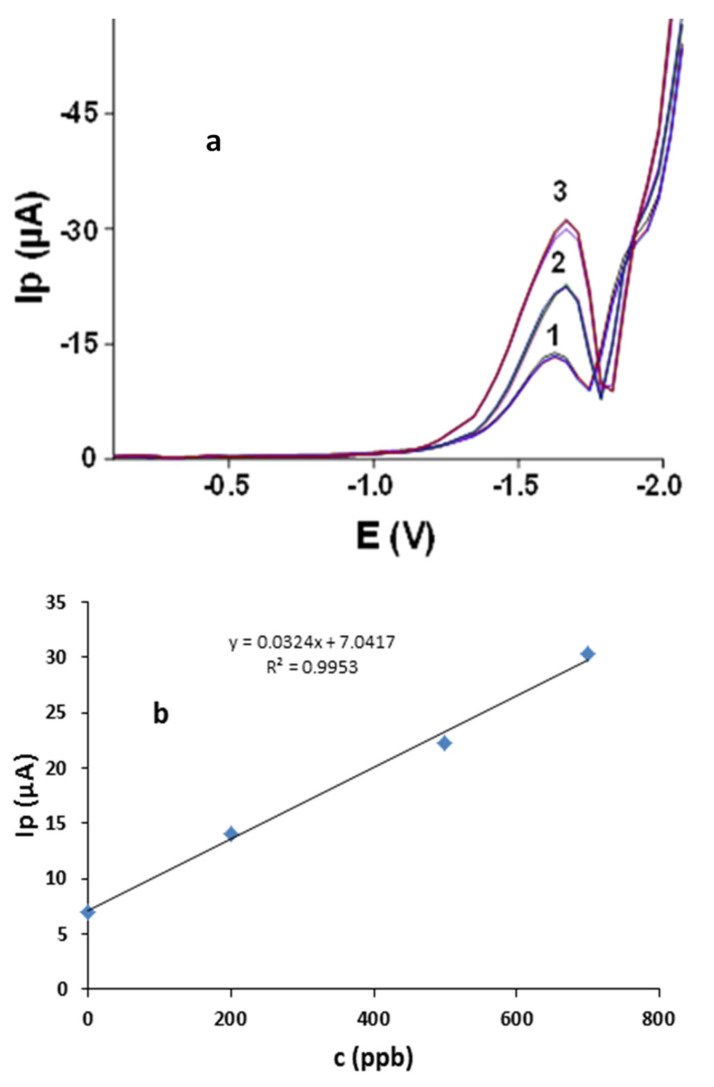
Three triple-performed SW voltammograms at Ibu-HgNPs-GCE of model local tap water sample with added 200, 500 and 700 ppb of AA under optimized conditions described in Section 3.2.9 (**a**) and corresponding calibration dependence (**b**).

Ibu-HgNPs-GCE was applied for SWV determination of AA in real samples of local tap water with added concentrations of 200 ppb, 500 ppb and 700 ppb AA (with triplicate runs) (see Figure 9 and Table 2). The range of recovery of AA was between 99.8–100.2%, with an average recovery of 99.6%. The recovery value of AA in the mentioned samples confirms the applicability of the Ibu-HgNPs-GCE sensor for monitoring of AA in water samples.

The determination of AA was also performed in the Indus River water samples (again in triplicate) obtained from various sites in Hyderabad, Pakistan, with added concentrations of 200 ppb, 500 ppb and 700 ppb AA (see Table 3). The recovery in this case was found to be from 98.3–101.0%. RSD in Indus River water is, however, higher than in tap water due to a more complex matrix. Nevertheless, Ibu-HgNPs-GCE is also applicable to these types of complex environmental aqueous matrices.

According to EPA, USA, the maximum contaminant level (MCL) in drinking water for a 10 kg child for one day of consumption is 1500 ppb, while for 10 days of consumption, it is 300 ppb. We worked on 300, 500 and 700 ppb and our developed method was justified and practically applicable below or above an MCL of 300 ppb. For further detail, refer to the reference information at https://nepis.epa.gov/Exe/ZyPDF.cgi/600012B6.PDF?Dockey=600012B6.PDF (accessed on 1 October 1995).

## 4. Conclusions

Mono-dispersed HgNPs were synthesized in an aqueous medium via hydrazine-assisted reduction and an ibuprofen capping protocol at room temperature, and characterized via UV-Vis spectroscopy and TEM. UV-Vis spectroscopy revealed the existence of an absorption band of Ibu-HgNPs at around 320 nm, while TEM analysis verified the existence of these Ibu-HgNPs in a solid form on the electrode surface with an average size of 3.5 ± 1.2 nm. Ibuprofen played a crucial role in the stabilization and size control of HgNPs. Nafion^TM^ deposition provided the best adherence and stabilization of Ibu-HgNPs at the surface of GCE and thus contributed as the ideal material for Ibu-HgNPs’ binding and long-term activity of modified GCE. Moreover, Nafion^TM^, as a perfluorinated polymeric resin possessing cation exchange properties with enhanced mechanical resistance, can work as an active fence. Due to cation exchange properties, the exchange of H^+^ ions on the polymer/electrode surface is increased, which gives rise to enhanced sensitivity in redox behavior. In addition, it also modifies the reproducibility and hence stability of the voltammetric responses. Due to such properties, Nafion is frequently used for modification of electrodes and thus we used it in this study as well [23].

Table 4 compares the results of AA determination using methods published so far [17,21,22,24,25,26] with the current study. It is evident that most of the electrochemical sensors are less sensitive when compared to our newly developed sensor. The most sensitive sensor reported so far is that in paper [25]. However, due to the use of several serial modifications, it is not cost-effective or simple.

Ibu-HgNPs deposited on GCE were used for the first time as highly sensitive and extremely selective modifiers for trace determination of AA in various water samples. The newly developed voltammetric AA sensor is simple and easy to construct, with a fast response, low cost and high stability, sensitivity and selectivity. Moreover, Ibu-HgNPs are more environmentally friendly than liquid mercury used in classical dropping mercury electrodes and/or hanging mercury electrodes, which are so far insurmountable working electrodes for the cathodic region. The portability of voltammetric equipment combined with this novel electrode and its application for on-site large-scale monitoring underlines the usefulness of this approach. Presumably, it can be used for highly selective and sensitive SWV determination of AA not only in environmental water samples but also in clinical, industrial, agricultural and other environmental samples. Further research is under way combining the newly introduced electrode with a preliminary separation and preconcentration from more complex samples.

## Figures and Tables

**Table 1:** Influence of possible interferents (each with 10 ppm concentration) on SWV determination of 1000 ppb AA under optimum conditions described in Section 3.2.9. Interference in % means the relative increase (+) or decrease (−) in SWV peak current of 1000 ppb AA (taken as 100%).

Interferent	Interference, %
K^+^	+1.2
Na^+^	+1.5
Ca^2+^	+2
NH_4_^+^	+2.6
Acetone	−1.1
Formaldehyde	−1.5
Acrolein	+0.7
Acrylic acid	+0.5
Maleic acid	−3.5
Glucose	−2.1
Citric acid	−3.4
Starch	+0

**Table 2:** SWV determination of AA at Ibu-HgNPs-GCE in local tap water under optimized conditions described in Section 3.2.9. Average from three determinations.

Local Water Sample	AA Added (ppb)	AA Found ± SD (ppb) *	Recovery (%)
1	0	- ^a^	
2	200	198 ± 0.6	99
3	500	501 ± 0.4	100.2
4	700	699 ± 1	99.8
			**Mean** 99.6

^a^ Below LOD of the proposed method; * calculated from three measurements.

**Table 3:** SWV determination of AA at Ibu-HgNPs-GCE in water of River Indus collected at three different sites with added 200, 500 and 700 ppb of AA. Optimized conditions described in Section 3.2.9. Average from three determinations.

Indus River Water	AA Added ^a^ (ppb)	AA Found ± SD (ppb)	Recovery (%)
Kotri site	150	148 ± 3.8	98.7
Al-Manzar site	300	295 ± 2.8	98.3
Jamshoro site	500	505 ± 4.1	101

^a^ In unspiked samples, AA concentration was below LOD of the proposed method.

**Table 4:** Comparison of published voltammetric methods for determination of acrylamide.

Technique	Electrode	Linear Range	LOD	Reference
DP polarography	DME	0.2–20 ppm	27 ppb	[17]
DPV	AuNPs-MWCNTs-CS-GCE	0.05–5 ppm	28 ppb	[21]
SWV	DNA/HG/SPGE	0.142–355.4 ppm	11.23 ppb	[22]
Polarography	DME	100–5000 ppm	70 ppm	[24]
DPV	SH-ssDNA-Au electrode	0.028–14216 ppm	0.58 ppb	[25]
Amperometry	Co-phthalocyanine modified SPE	0.5–3500 ppm	355 ppb	[26]
SWV	HgNPs-GCE	0.1–1.3 ppm	8.5 ppb	This work

LOD, limit of detection; DPV, differential pulse voltammetry; SWV, square-wave voltammetry; DME, dropping mercury electrode; MWCNTs, multi walled carbon nanotubes; HG, hemoglobin; SPGE, screen printed gold electrode; SH-ssDNA, Thio group-single strand DNA.

## Data Availability

Not applicable.
